# A Comparison of Higher-Level Functional Capacity Between Older Adults with and Without Type 2 Diabetes Mellitus: A Cross-Sectional Study Using Propensity Score Matching

**DOI:** 10.3390/geriatrics10050115

**Published:** 2025-08-26

**Authors:** Takuro Shoji, Kenta Kogure, Nagisa Toda, Mariko Hakoshima, Hisayuki Katsuyama, Hidekatsu Yanai, Satoshi Tokunaga, Korin Tateoka, Taishi Tsuji, Tomohiro Okura

**Affiliations:** 1 Department of Rehabilitation Medicine, National Center for Global Health and Medicine Kohnodai Hospital, Ichikawa 272-8516, Chiba, Japan; kogure.k@jihs.go.jp (K.K.); toda.n@jihs.go.jp (N.T.); 2 Doctoral Program in Public Health, Graduate School of Comprehensive Human Sciences, University of Tsukuba, Tuskuba 305-8574, Ibaraki, Japan; immaturofantasista@gmail.com; 3 Department of Diabetes, Endocrinology and Metabolism, National Center for Global Health and Medicine Kohnodai Hospital, Ichikawa 272-8516, Chiba, Japan; hakoshima.m@jihs.go.jp (M.H.); katsuyama.h@jihs.go.jp (H.K.); yanai.h@jihs.go.jp (H.Y.); 4 Doctoral Program in Physical Education, Graduate School of Comprehensive Human Sciences, Health and Sport Sciences, University of Tsukuba, Tuskuba 305-8574, Ibaraki, Japan; korin4416@gmail.com; 5 Center for Preventive Medical Science, Chiba University, Chiba 263-8522, Chiba, Japan; tsuji.taishi.gn@u.tsukuba.ac.jp; 6 Institute of Health and Sport Sciences, University of Tsukuba, Tuskuba 305-8574, Ibaraki, Japan; okura.tomohiro.gp@u.tsukuba.ac.jp

**Keywords:** intellectual activity, Japan science and technology agency index of competence, social engagement, Tokyo metropolitan institute of gerontology index of competence, type 2 diabetes mellitus

## Abstract

*Background/Objectives*: In Japan, the number of older patients with diabetes mellitus (DM) is rapidly increasing; however, the impact of DM on higher-level functional capacity in this population is unclear. In this study, we aimed to clarify the characteristics of higher functional capacity in older patients with type 2 diabetes mellitus (T2DM). *Methods*: The participants included outpatients with T2DM receiving care at a general hospital and community-dwelling older adults without DM (both groups aged ≥ 65 years) in Japan. The Tokyo Metropolitan Institute of Gerontology Index of Competence (TMIG-IC) and the Japan Science and Technology Agency Index of Competence (JST-IC) were used to evaluate higher-level functional capacity. We compared the higher-level functional capacities of the two groups after propensity score matching to ensure homogeneity in background factors. *Results*: After propensity score matching, 131 individuals each from a group of older patients with T2DM and a group of community-dwelling older adults without DM were included (mean age: 76.6 ± 5.6 and 76.1 ± 5.4 years, respectively; male sex: 54.2% and 52.7%, respectively). The older patients with T2DM had higher average instrumental activities of daily living scores (4.8 vs. 4.6; *p* < 0.01) and lower average intellectual activity scores (3.4 vs. 3.8; *p* < 0.01) on the TMIG-IC, average JST-IC scores (10.3 vs. 11.6; *p* < 0.01), and average social engagement scores (1.0 vs. 2.2; *p* < 0.01) compared to the community-dwelling older adults without DM. *Conclusions*: Older outpatients with T2DM demonstrated poorer intellectual activity and social engagement than community-dwelling older adults without DM. Therefore, it may be necessary to focus on preventive interventions to support higher-level functional capacities in this population.

## 1. Introduction

In Japan, there are 10 million adults in whom diabetes mellitus (DM) is strongly suspected, who are undergoing treatment for DM, who have a hemoglobin A1c (HbA1c) level of 6.5% or more, or for whom the possibility of DM cannot be ruled out (HbA1c: 6.0–6.5%) [1]. Furthermore, the number of older patients with DM is swiftly increasing due to Japan’s rapidly aging population [2]. As various adverse events that affect self-care are likely to occur in older patients with DM, maintaining functional capacity (functional health) is important in this population.

The World Health Organization proposes that functional capacity, that is, the ability to perform the comprehensive functions necessary for older adults to live independently, be used as a health indicator in this population [3]. Lawton systematizes functional capacity into the following seven levels, ranging from simple to complex functions: life maintenance, functional health, perception–cognition, physical self-maintenance, instrumental independence, intellectual activity, and social roles [4]. In this model, the three upper levels, instrumental independence, intellectual activity, and social roles, represent higher-level functional capacity, constituting the abilities that older adults need to live independently in their community [5]. Poor higher-level functional capacity is associated with an increased risk of death, disability, and stroke and higher medical and nursing care costs [6,7,8]. Therefore, maintaining higher-level functional capacity may contribute to reducing the risk of future disability or death and medical and nursing care costs.

Reduced higher-level functional capacity is characterized by a gradual decline in complex functions relating to social roles, intellectual activity, and instrumental activities of daily living (IADL) [9]. In addition, a systematic review found that older adults with DM have a high risk of developing disabilities [10]. Therefore, older patients with DM may have more higher-level functional capacity impairments than those without DM. Although some studies have investigated higher-level functional capacity in older patients with DM in single groups [11,12,13,14], to the best of our knowledge, no study has established a control group to comparatively examine higher-level functional capacity. Therefore, the impact of DM on higher-level functional capacity in older patients is unclear.

In Japan, the Tokyo Metropolitan Institute of Gerontology Index of Competence (TMIG-IC) is widely used to quantify higher-level functional capacity [15,16]. However, due to recent changes in the health conditions, living environments, and lifestyles of older people, the TMIG-IC alone is insufficient for evaluating higher-level functional capacity in this population [5]. In fact, a study conducted on community-dwelling older adults reported a ceiling effect in the TMIG-IC [17]. Iwasa et al. developed the Japan Science and Technology Agency Index of Competence (JST-IC), which corresponds better to the current lifestyles of older individuals and makes it possible to quantify aspects of higher-level functional capacity that the TMIG-IC cannot capture accurately [5,18]. The combined use of the TMIG-IC and JST-IC enables a detailed and multifaceted evaluation of higher-level functional capacity. However, the characteristics of higher-level functional capacity in older patients with type 2 diabetes mellitus (T2DM), evaluated using the JST-IC, remain unclear.

Therefore, in this study, we aimed to clarify the characteristics of higher-level functional capacity, using the TMIG-IC and JST-IC, in older patients with T2DM by comparing them with community-dwelling older adults without DM. Clarifying the areas of reduced higher-level functional capacity in older patients with T2DM may enable the provision of focused preventive interventions or provide support for a decline in higher-level functional capacity. We hypothesized that older patients with T2DM have a poorer overall higher-level functional capacity than community-dwelling older adults without DM, particularly with respect to social roles and intellectual activity (i.e., more complex functions).

## 2. Materials and Methods

### 2.1. Study Design and Participants

**Figure 1:**
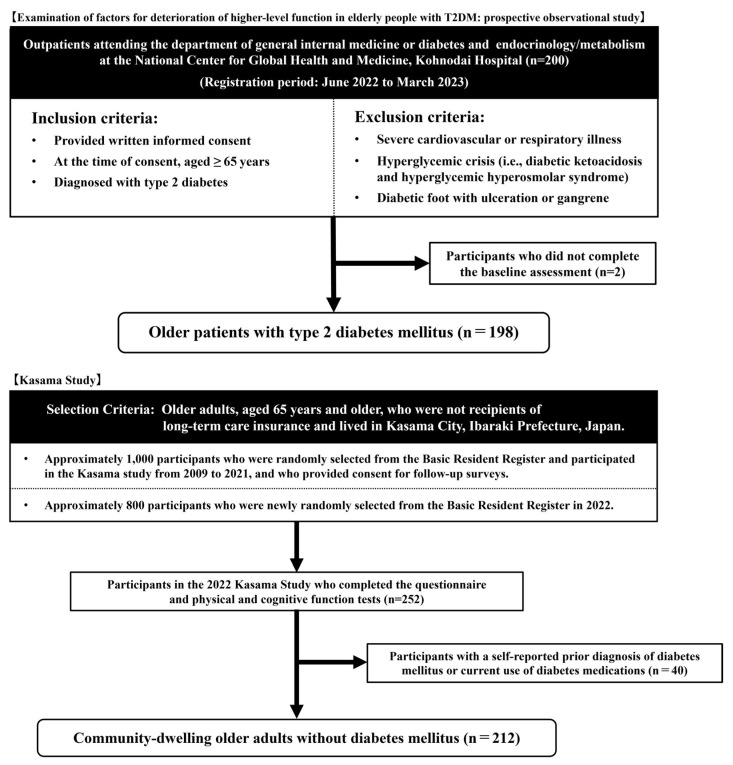
Flowchart illustrating the recruitment process for the study “Examination of factors for deterioration of higher-level function in elderly people with T2DM: prospective observational study” and the “Kasama study”.

The participants in this study were drawn from two cohorts (Figure 1). The group of older adults with T2DM comprised participants from a cohort study (“Examination of factors for deterioration of higher-level function in elderly people with T2DM: prospective observational study”) [19] conducted at the National Center for Global Health and Medicine Kohnodai Hospital (Ichikawa City, Chiba Prefecture, Japan), from June 2022 to March 2023. A total of 200 participants were enrolled in the study. The inclusion criteria for this cohort were outpatients aged 65 years or older with a diagnosis of T2DM who provided written informed consent. Participants were excluded if they had severe cardiovascular or respiratory diseases, hyperglycemic crises (i.e., diabetic ketoacidosis or hyperglycemic hyperosmolar syndrome), or diabetic foot with ulceration or gangrene. Among those enrolled, 198 participants who completed the baseline evaluations were included in this analysis. Community-dwelling older adults without diabetes mellitus (DM) were drawn from the Kasama Study [20], which was conducted in September 2022. The participants in the Kasama Study were community-dwelling adults aged 65 years or older who were not certified as requiring long-term care and resided in Kasama City, Ibaraki Prefecture, Japan. Approximately 1800 individuals were randomly selected from the Basic Resident Register and invited to participate in a health checkup. Of those invited, 252 individuals who wished to participate underwent assessments of physical function, cognitive function, and psychological and social environmental factors at the health checkup site. On the questionnaire survey, 40 individuals who answered “yes” to either of the following questions, “Have you ever been diagnosed with diabetes mellitus?” or “Are you currently receiving treatment for diabetes mellitus?”, were excluded. Consequently, 212 individuals were included in this analysis as community-dwelling older adults without diabetes.

This study was approved by the ethics board of the National Center for Global Health and Medicine (approval number: NCGM-S-004741-00) and was conducted in accordance with the Declaration of Helsinki. As this was a retrospective study that used existing data, informed consent was obtained through an opt-out protocol. The opportunity to opt out was provided on the website of the Okura Laboratory, Institute of Health and Sport Sciences, University of Tsukuba, and necessary information was posted on the bulletin board of the National Center for Global Health and Medicine Kohnodai Hospital.

### 2.2. Assessment of Higher-Level Functional Capacity

Self-administered questionnaires containing the TMIG-IC [15,16] and JST-IC [5,18] were used to evaluate higher-level functional capacity in the study population (Supplementary Figure S1). The community-dwelling older adults without diabetes completed the assessment questionnaire at the time of their participation in the health checkup program, while the older adults with type 2 diabetes completed the questionnaire during their first outpatient visit after providing informed consent to participate in the study. The TMIG-IC assesses three areas (IADL, intellectual activity, and social roles), whereas the JST-IC evaluates four areas (technology usage, information practice, life management, and social engagement). Each question was answered with either yes (1 point) or no (0 point), with higher scores indicating a better condition (score range: 0–13 points for the TMIG and 0–16 points for the JST-IC). The TMIG-IC has a high reliability and validity (Cronbach’s α coefficient = 0.913) [15]. The JST-IC is more complex than the TMIG-IC and can measure higher-level functional capacity (construct validity), and its reliability and validity have been reported (Cronbach’s α coefficient = 0.86) [5,18]. We calculated the total score, subscale scores, and percentage of respondents who answered “no” to each question for both scales.

### 2.3. Other Variables

The following information was used to calculate the propensity score: sex, age, body mass index (BMI), presence of chronic diseases (hypertension, cardiovascular disease, and cerebrovascular disease), smoking and drinking habits, self-rated health, economic conditions, living status (living alone or not), years of education, and physical activity. BMI was calculated from the participants’ height and weight. The presence of chronic diseases (hypertension, cardiovascular disease, and cerebrovascular disease) was determined if the participants answered “yes” to “have been diagnosed in the past” or “currently receiving treatment”. The Physical Activity Scale for the Elderly (PASE) was used to assess physical activity [21]. The PASE consists of leisure-, home-, and work-related activities, and the sum of these is the total physical activity score (PASE score). A high PASE score indicates a high level of physical activity.

To characterize older patients with T2DM, we collected information on their HbA1c levels, diabetic complications (neuropathy, retinopathy, and nephropathy), and medication use. Neuropathy was defined as the fulfillment of at least two of the following criteria: the presence of subjective symptoms (numbness, pain, and paresthesia in both soles or toes), reduced Achilles tendon reflex, and decreased vibration sensation in the medial malleolus [22]. Retinopathy was classified based on the Davis classification [23], and patients with simple retinopathy, pre-proliferative retinopathy, or proliferative retinopathy were defined as having retinopathy in this study. Nephropathy was classified based on the results of the urinary albumin/creatinine ratio and estimated glomerular filtration rate according to the Diabetic Nephropathy 2014 classification [24]. Patients with nephropathy were defined as those with nephropathy stage 2 (early nephropathy) or higher.

### 2.4. Statistical Analysis

High-quality propensity score matching requires sufficient data on baseline variables shared by both cohorts. As many such variables were available in this study, we used data from both cohorts to ensure comparability between groups.

We used Pearson’s chi-square test for categorical variables and the unpaired t-test for continuous variables to compare the characteristics of the two cohorts before matching. Propensity score matching was performed to homogenize the characteristics of the two cohorts. Propensity score estimates were determined using logistic regression analysis, with the presence of DM as the dependent variable and all variables investigated to calculate the propensity score as independent variables. To determine the discriminative power of the propensity score, the receiver operating characteristic (ROC) curve, with the propensity score as the test variable, was used, and the area under the curve (AUC) was calculated.

We used nearest-neighbor matching, which extracts pairs with similar propensity scores from both groups on a one-to-one basis. The absolute value of the propensity score of the extracted pair was required to be within a certain caliper (threshold), which was set at 0.2 times the standard deviation (SD) of the propensity score for all participants [25]. To confirm the balance of characteristics between both groups pre- and post-matching, the standardized difference (d), an indicator of balance, was calculated using the formula mentioned below. In this study, a standardized difference of less than 0.1 was used as a criterion for determining the homogeneity of the characteristics of the groups [26].

[For categorical variables]

(1) $$d=\frac{\left|P_{A}-P_{B}\right|}{\sqrt{\frac{P_{A}(1-P_{A})+P_{B}(1-P_{B})}{2}}}$$

*P_A_* and *P_B_* are the percentages of categorical variables in older patients with T2DM (A) and community-dwelling older adults without DM (B), respectively.

[For continuous variables]

(2) $$d=\frac{\left|{\bar{x}}_{A}-{\bar{x}}_{B}\right|}{\sqrt{\frac{{S_{A}}^{2}+{S_{B}}^{2}}{2}}}$$

${\bar{x}}_{A}$ and ${\bar{x}}_{B}$ are the mean values of continuous variables in older patients with T2DM (A) and community-dwelling older adults without DM (B), respectively.

${S_{A}}^{2}$ and ${S_{B}}^{2}$ represent the variance in continuous variables in older patients with T2DM (A) and community-dwelling older adults without DM (B), respectively.

After confirming the homogeneity of the characteristics in both groups, we compared the higher-level functional capacity of the two cohorts. The Mann–Whitney U test was used to compare the total scores and scores for each subscale on the TMIG-IC and JST-IC. Pearson’s chi-square test was used to compare the proportion of participants who answered “no” to each question.

All statistical analyses were performed using IBM SPSS version 28.0 (IBM Corp., Armonk, NY, USA), and a two-tailed *p*-value of less than 0.05 was considered statistically significant.

## 3. Results

### 3.1. Discriminative Power of Propensity Score

In this study, the AUC of the ROC curve with the propensity score as the test variable was 0.736. As the AUC must be between 0.6 and 0.9, this outcome confirms that the discriminative power of the propensity score was adequate.

### 3.2. Participants’ Characteristics Pre- and Post-Propensity Score Matching

Table 1 and Table 2 present the participants’ characteristics. Pre-matching, the mean (SD) ages of the older patients with T2DM and community-dwelling older adults without DM were 75.9 (5.7) years and 76.3 (5.3) years, respectively. The older patients with T2DM had a higher BMI and lower PASE score than the community-dwelling older adults without DM. Furthermore, the older patients with T2DM were more likely to be male, be hypertensive, have cerebrovascular disease, be current smokers, have poor self-rated health, have poor economic conditions, and live alone. After propensity score matching, there were 131 participants in each group. Standardized differences were less than 0.1 for all variables used to calculate the propensity scores. Therefore, the homogeneity of characteristics was confirmed in both groups. In older patients with T2DM, the mean (SD) HbA1c level was 7.1% (0.9%), and the prevalence rates of diabetic neuropathy, retinopathy, and nephropathy were 38.9%, 26.0%, and 42.7%, respectively.

### 3.3. Comparison of Scores on the Higher-Level Functional Capacity Index

**Figure 2:**
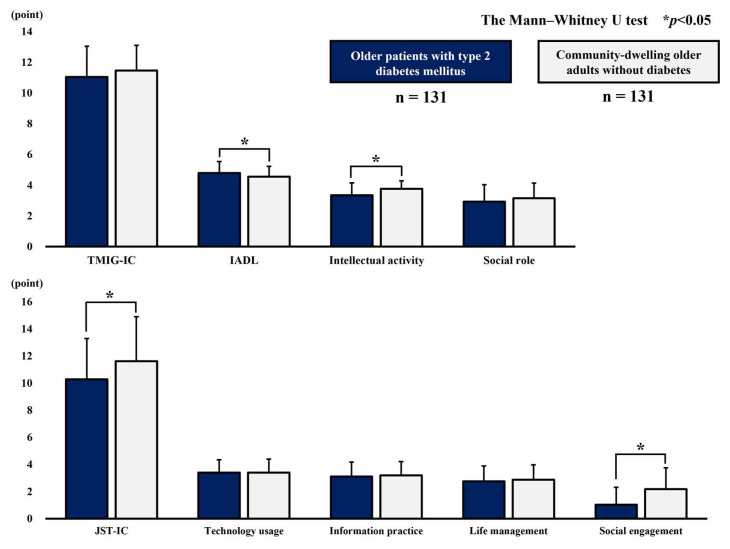
Comparison of scores on higher-level functional capacity indices (TMIG: top; JST: bottom). TMIG-IC, Tokyo Metropolitan Institute Gerontology Index of Competence; IADL, instrumental activities of daily living; JST-IC, Japan Science and Technology Agency Index of Competence.

Table 3 and Figure 2 show the total and subscale scores of the TMIG-IC and JST-IC in the two cohorts. On the TMIG-IC, the older patients with T2DM had significantly higher IADL scores (4.8 vs. 4.6 points) and lower intellectual activity scores (3.4 vs. 3.8 points) than the community-dwelling older adults without DM. On the JST-IC, the older patients with T2DM had significantly lower JST-IC total scores (10.3 vs. 11.6 points) and social engagement scores (1.0 vs. 2.2 points) than the community-dwelling older adults without DM.

### 3.4. Comparison of the Percentage of Participants Reporting Difficulty with Each Item of the Higher-Level Functional Capacity Index

**Figure 3:**
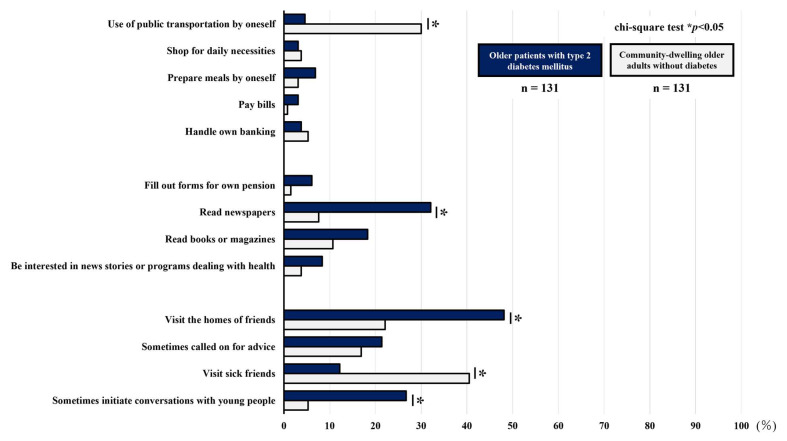
Comparison the percentage of participants reporting difficulty with each question on the TMIG-IC.

**Figure 4:**
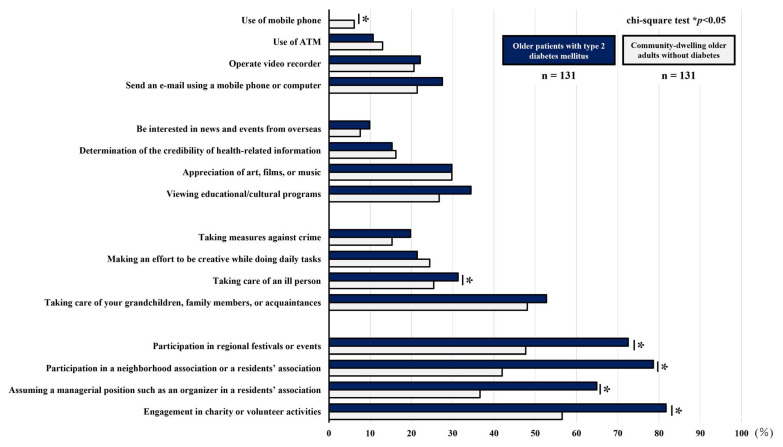
Comparison of the percentage of participants reporting difficulty with each question on the JST-IC.

Table 4 and Table 5 and Figure 3 and Figure 4 compare the percentages of participants reporting difficulty with each item in the TMIG-IC and JST-IC. The older patients with T2DM were more likely than the community-dwelling older adults without DM to report difficulties with the following items: “read newspapers”, “visit the homes of friends”, and “sometimes initiate conversations with young people” in the TMIG-IC and all items of the social engagement subscale in the JST-IC. However, the community-dwelling older adults without DM were more likely than the older patients with T2DM to report difficulties with the following items: “use of public transportation by oneself” and “visit sick friends” in the TMIG-IC and “use of mobile phone” in the JST-IC.

## 4. Discussion

We compared higher-level functional capacity, which was assessed using the TMIG-IC and JST-IC, in older outpatients with T2DM who were receiving care at a general hospital and community-dwelling older adults without DM. The comparison was conducted after propensity score matching to homogenize the characteristics of the groups. Consequently, the older patients with T2DM had significantly lower scores for intellectual activity on the TMIG-IC and total scores and social engagement on the JST-IC than the community-dwelling older adults without DM. Moreover, the older patients with T2DM were more likely to report difficulties with social roles and engagement than the community-dwelling older adults without DM. These results support our hypothesis and clarify that older patients with T2DM can be characterized by a decline in more complex higher-level functional capacities. However, contrary to our hypothesis, the IADL scores were significantly lower in the community-dwelling older adults without DM than in the older patients with T2DM. Nevertheless, our findings may provide useful information regarding targets for intensive preventive interventions to combat the decline in higher-level functional capacity in older patients with T2DM.

Regarding the propensity score matching method used in this study, the AUC of the ROC curve with the propensity score as the test variable was 0.736. An AUC must be between 0.6 and 0.9; hence, the discriminative power of the propensity score was adequate. In addition, standardized differences were less than 0.1 for all variables used to calculate the propensity scores, confirming the homogeneity of the characteristics in both groups.

In this study, older patients with T2DM were characterized by a decline in more complex higher-level functional capacities (i.e., intellectual activity, social engagement, and JST-IC subscales) than community-dwelling older adults without DM. This result is reasonable considering the sequential nature of the decline in higher-level functional capacity (i.e., complex functional capacity tends to decline first) [9]. Moreover, the JST-IC is a more detailed indicator of higher-level functional capacity than the TMIG-IC [18,27].

The National Health and Nutrition Examination Survey, conducted in the United States, reported that older adults with DM have difficulty engaging in leisure and social activities (such as going to movies, shopping, and engaging in social activities) compared with those without DM [28]. Although the evaluation indicators of social roles and social engagement were different, the results of this study are similar to those of previous studies. Cross-sectional associations have been reported between diminished social function and the presence of diabetic nephropathy, depression, and frailty in older patients with DM [29]. Diabetes-related complications and geriatric syndromes may have a negative impact on social roles and social engagement in older patients with DM. In this study, many older patients with DM reported difficulties with intellectual activities, particularly reading the newspaper. As life expectancy is increasing, the number of older patients with visual impairment due to diabetic retinopathy is also increasing [30]. In this study, diabetic retinopathy was observed in 26.0% of older patients with DM. The progression of diabetic retinopathy can cause symptoms such as blurred or hazy vision. Intellectual activities include tasks such as preparing documents and reading newspapers, books, or magazines, which require the ability to focus on small print. Therefore, the presence of diabetic retinopathy may potentially contribute to a decline in the capacity for these activities. These findings suggest that impaired visual function could be a factor leading to decreased intellectual engagement in older patients with DM. Based on the above, promoting intellectual activity and social participation is essential for older adults with type 2 diabetes, and community-based programs may offer specific benefits for this population. For example, digital literacy workshops can support cognitive engagement, while neighborhood social clubs can provide opportunities for social interaction. Such locally tailored interventions may help reduce social isolation, maintain cognitive function, and ultimately improve quality of life in this population.

Contrary to our hypothesis, the community-dwelling older adults without type 2 diabetes (T2DM) had significantly lower IADL scores than the participants with T2DM. In particular, a greater proportion of individuals without T2DM reported difficulties in using public transportation independently and visiting sick friends compared to those with T2DM. Previous studies have shown that older adults living in suburban or rural areas tend to have lower IADL scores than those in urban areas [31]. Although we used propensity score matching to homogenize the characteristics of the older adults with T2DM and those without, geographic and environmental factors may not have been adequately accounted for. In this study, the community-dwelling older adults without diabetes were sampled from a rural area, which may partially explain their lower IADL capacity compared to the general population of older adults in Japan. In contrast, the older adults with T2DM were recruited from a hospital in a suburban area near the capital, where residents generally have better access to public transportation, medical facilities, ATMs, and other essential services. This regional context may have contributed to their relatively higher functional capacity compared to the broader population of older adults with T2DM in Japan. Therefore, the observed difference in IADL capacity between the two cohorts may be underestimated (i.e., older adults with diabetes tend to exhibit a lower IADL ability, while community-dwelling older adults tend to have a higher IADL ability). These differences in residential areas may have influenced the IADL scores, particularly in the domains related to the independent use of public transportation and social visits. Additionally, contrary to our hypothesis, none of the older patients with T2DM reported difficulty with the use of mobile phones. The University of Michigan reported that approximately one in four health application users among older adults with T2DM use these applications to control their blood sugar [32]. Hence, older patients with DM often use mobile devices to manage their disease and collect health information, potentially making them more familiar with mobile devices. In addition, increased access to medical services (frequency of hospital visits) may have led to more frequent use of ICT devices for purposes such as communicating with family and friends, checking public transportation schedules, and searching for transfer information. This increased use of ICT devices may have contributed to the maintenance or improvement of technological skills among the older adults with type 2 diabetes.

The strengths of our study are that it included a control group and extensively clarified the characteristics of higher-level functional capacity, which was assessed multidimensionally using the TMIG-IC and JST-IC, in older patients with T2DM. However, this study also had a few limitations. First, as this was a cross-sectional study, we were unable to establish a causal relationship between DM and higher-level functional capacity. Further studies are required to clarify the causal inference between these two factors, including longitudinal investigations and interventional research. Second, sampling bias was present because the two cohorts were drawn from different regions. Differences in geographic location and environmental factors may have influenced the IADL scores. To address this issue, it is necessary to conduct sampling across multiple institutions and regions or to obtain detailed information on local environments in order to incorporate environmental factors into the analysis. Third, the study sample consisted exclusively of older adults in Japan, and the instrument used to assess higher-level functional capacity was also developed in Japan. Therefore, the generalizability of our findings to countries with different cultural backgrounds and lifestyles may be limited. In particular, the JST-IC subscales “Technology Usage” and “Information Practice” may be strongly influenced by societal factors such as national or regional educational systems, the prevalence of ICT devices, and economic conditions. Thus, caution is warranted when applying these assessment tools or generalizing the results of this study to different populations. Fourth, we determined the control group (community-dwelling older adults without DM) based on the participants’ answers (i.e., if they answered “no” to “have been diagnosed with diabetes mellitus in the past” or “currently receiving treatment for diabetes mellitus”). HbA1c and blood glucose levels were not assessed in the control group; therefore, diabetes could not be excluded based on standard diagnostic criteria, and participants with undiagnosed diabetes mellitus may have been inadvertently included. In future research, it will be important to obtain diagnostic data based on standard criteria for diabetes in the control group so that individuals with undiagnosed diabetes can be excluded and the characteristics of the group are more clearly defined. Finally, the older patients with T2DM in our study were outpatients receiving care at a general hospital; therefore, the results may not apply to hospitalized or institutionalized patients. Future studies with larger target populations are warranted.

In conclusion, older patients with T2DM showed better scores for some IADL items (use of public transportation by oneself), but worse scores for more complex higher-level functional capacity (social engagement, social roles, and intellectual activity) items than community-dwelling older adults without DM. Therefore, focused preventive intervention and support for social engagement, social roles, and intellectual activity may reduce the risk of future disability or death in older patients with T2DM.

## Figures and Tables

**Table 1:** Characteristics of participants pre- and post-propensity score matching.

	Pre-Propensity Score Matching								Post-Propensity Score Matching						
	Older Patients with Type 2 Diabetes Mellitus (n = 198)			Community-Dwelling Older Adults Without Diabetes (n = 212)			*p*	Std Diff	Older Patients with Type 2 Diabetes Mellitus (n = 131)			Community-Dwelling Older Adults Without Diabetes (n = 131)			Std Diff

	Mean	±	SD	Mean	±	SD			Mean	±	SD	Mean	±	SD	
Male sex, n (%)	119 (60.1)			90 (42.5)			<0.01	0.36	71 (54.2)			69 (52.7)			0.03
Age, years	75.9	±	5.7	76.3	±	5.3	0.47	0.07	76.6	±	5.6	76.1	±	5.4	0.09
BMI, kg/m^2^	24.6	±	3.9	23.0	±	3.1	<0.01	0.47	23.7	±	3.4	23.5	±	3.1	0.06
Hypertension, n (%)	104 (52.5)			87 (41.0)			0.02	0.23	61 (46.6)			58 (44.3)			0.04
Cardiovascular disease, n (%)	20 (10.1)			12 (5.7)			0.10	0.16	10 (7.6)			7 (5.3)			0.09
Cerebrovascular disease, n (%)	19 (9.6)			6 (2.8)			<0.01	0.28	6 (4.6)			6 (4.6)			0
Current smoker, n (%)	23 (11.6)			7 (3.3)			<0.01	0.32	6 (4.6)			7 (5.3)			0.03
Alcohol consumption (drinker), n (%)	52 (26.4)			61 (28.8)			0.65	0.05	41 (31.3)			37 (28.2)			0.06
Poor self-rated health, n (%)	32 (16.2)			17 (8.0)			0.01	0.25	14 (10.7)			13 (9.9)			0.02
Poor economic conditions, n (%)	45 (22.7)			29 (13.7)			0.02	0.23	23 (17.6)			24 (18.3)			0.01
Living alone, n (%)	45 (22.7)			31 (14.6)			0.04	0.21	22 (16.8)			21 (16.0)			0.02
Period of education, years	13.3	±	2.7	12.9	±	2.3	0.71	0.18	13.1	±	2.8	13.0	±	2.5	0.03
Physical activity (PASE score), point	103.5	±	66.6	123.1	±	50.3	<0.01	0.33	116.6	±	65.5	113.5	±	43.1	0.05

SD, standard deviation; Std diff, standardized difference; BMI, body mass index; PASE, Physical Activity Scale for the Elderly.

**Table 2:** Characteristics of older patients with type 2 diabetes mellitus post-propensity score matching (n = 131).

	n	Mean	±	SD
HbA1c, %	130	7.1	±	0.9
Neuropathy, n (%)	131	51 (38.9)		
Retinopathy, n (%)	112	34 (26.0)		
Nephropathy, n (%)	130	56 (42.7)		
Use of medication				
Insulin	131	29 (22.1)		
Sulfonylureas	131	15 (11.5)		
Biguanides	131	73 (55.7)		
Thiazolidines	131	18 (13.7)		
α-Glucosidase inhibitors	131	21 (16.0)		
Glinides	131	14 (10.7)		
DPP-4 inhibitors	131	83 (63.4)		
SGLT2 inhibitors	131	66 (50.4)		
GLP-1 receptor agonists	131	24 (18.3)		

SD, standard deviation; HbA1c, hemoglobin A1c; DPP-4. dipeptidyl peptidase 4; SGLT2, sodium-glucose cotransporter-2; GLP-1, glucagon-like peptide-1.

**Table 3:** Comparison of TMIG-IC and JST-IC scores between older patients with type 2 diabetes mellitus (n = 131) and community-dwelling older adults without diabetes (n = 131).

	Older Patients with Type 2 Diabetes Mellitus			Community-Dwelling Older Adults Without Diabetes			*p*
	(n = 131)			(n = 131)			
	Mean	±	SD	Mean	±	SD	
TMIG-IC score, point	11.1	±	2.0	11.5	±	1.6	0.07
IADL score, point	4.8	±	0.8	4.6	±	0.7	<0.01
Intellectual activity score, point	3.4	±	0.8	3.8	±	0.5	<0.01
Social role score, point	2.9	±	1.1	3.2	±	1.0	0.10
JST-IC score, point	10.3	±	3.0	11.6	±	3.3	<0.01
Technology usage score, point	3.4	±	0.9	3.4	±	1.0	0.90
Information practice score, point	3.1	±	1.1	3.2	±	1.0	0.47
Life management score, point	2.8	±	1.1	2.9	±	1.1	0.38
Social engagement score, point	1.0	±	1.3	2.2	±	1.6	<0.01

SD, standard deviation; TMIG-IC, Tokyo Metropolitan Institute Gerontology Index of Competence; IADL, instrumental activities of daily living; JST-IC, Japan Science and Technology Agency Index of Competence.

**Table 4:** Comparison of the percentage of participants reporting difficulty with each question of the TMIG-IC between older patients with type 2 diabetes mellitus (n = 131) and community-dwelling older adults without diabetes (n = 131).

	Older Patients with Type 2 Diabetes Mellitus	Community-Dwelling Older Adults Without Diabetes	*p* *
	(n = 131)	(n = 131)	
	Percentage of Older Adults with Difficulty, n (%)	Percentage of Older Adults with Difficulty, n (%)	
IADL			
Use of public transportation by oneself	6 (4.6)	39 (30.0)	<0.01
Shop for daily necessities	4 (3.1)	5 (3.8)	0.73
Prepare meals by oneself	9 (6.9)	4 (3.1)	0.16
Pay bills	4 (3.1)	1 (0.8)	0.18
Handle own banking	5 (3.8)	7 (5.3)	0.55
Intellectual activity			
Fill out forms for own pension	8 (6.1)	2 (1.5)	0.05
Read newspapers	42 (32.1)	10 (7.6)	<0.01
Read books or magazines	24 (18.3)	14 (10.7)	0.08
Be interested in news stories or programs dealing with health	11 (8.4)	5 (3.8)	0.12
Social role			
Visit the homes of friends	63 (48.1)	29 (22.1)	<0.01
Sometimes called on for advice	28 (21.4)	22 (16.9)	0.36
Visit sick friends	16 (12.2)	53 (40.5)	<0.01
Sometimes initiate conversations with young people	35 (26.7)	7 (5.3)	<0.01

* chi-square test. IADL, instrumental activities of daily living; TMIG-IC, Tokyo Metropolitan Institute Gerontology Index of Competence.

**Table 5:** Comparison of the percentage of participants reporting difficulty with each question on the JST-IC between older patients with type 2 diabetes mellitus (n = 131) and community-dwelling older adults without diabetes (n = 131).

	Older Patients with Type 2 Diabetes Mellitus	Community-Dwelling Older Adults Without Diabetes	*p* *
	(n = 131)	(n = 131)	
	Percentage of Older Adults with Difficulty, n (%)	Percentage of Older Adults with Difficulty, n (%)	
Technology usage			
Use of mobile phone	0 (0)	8 (6.1)	<0.01
Use of ATM	14 (10.7)	17 (13.0)	0.57
Operate video recorder (Blu-ray recorder or DVD player)	29 (22.1)	27 (20.6)	0.76
Send an e-mail using a mobile phone or computer	36 (27.5)	28 (21.4)	0.25
Information practice			
Be interested in news and events from overseas	13 (9.9)	10 (7.6)	0.51
Determination of the credibility of health-related information	20 (15.3)	21 (16.2)	0.84
Appreciation of art, films, or music	39 (29.8)	39 (29.8)	1.00
Viewing educational/cultural programs	45 (34.4)	35 (26.7)	0.18
Life management			
Taking measures against crime	26 (19.8)	20 (15.3)	0.33
Making an effort to be creative while doing daily tasks	28 (21.4)	32 (24.4)	0.56
Taking care of an ill person	41 (31.3)	33 (25.4)	0.29
Taking care of your grandchildren, family members, or acquaintances	69 (52.7)	63 (48.1)	0.46
Social engagement			
Participation in regional festivals or events	95 (72.5)	62 (47.7)	<0.01
Participation in a neighborhood association or a residents’ association	103 (78.6)	55 (42.0)	<0.01
Assuming a managerial position such as an organizer in a residents’ association	85 (64.9)	48 (36.6)	<0.01
Engagement in charity or volunteer activities	107 (81.7)	74 (56.5)	<0.01

* chi-square test. ATM, Automatic Teller Machine; DVD, digital versatile disc; JST-IC, Japan Science and Technology Agency Index of Competence.

## Data Availability

The data are available on request due to privacy, legal, and ethical restrictions. The data that support the findings of this study are available from the corresponding author upon reasonable request. The data are not publicly available due to privacy or ethical restrictions.

## Supplementary Materials

The following supporting information can be downloaded at: https://www.mdpi.com/article/10.3390/geriatrics10050115/s1, Figure S1: Indices of higher-level functional capacity (TMIG-IC, JST-IC).

## Abbreviations

The following abbreviations are used in this manuscript:

DM	diabetes mellitus
HbA1c	hemoglobin A1c
IADL	instrumental activities of daily living
TMIG-IC	Tokyo Metropolitan Institute of Gerontology Index of Competence
JST-IC	Japan Science and Technology Agency Index of Competence
T2DB	type 2 diabetes mellitus
BMI	Body Mass Index
PASE	Physical Activity Scale for the Elderly
ROC	receiver operating characteristic
AUC	area under the curve
SD	standard deviation
