# Supplementary material for: A Comparison of Higher-Level Functional Capacity Between Older Adults with and Without Type 2 Diabetes Mellitus: A Cross-Sectional Study Using Propensity Score Matching
Source: Geriatrics (Basel). 2025 Aug 26;10(5):115. doi: 10.3390/geriatrics10050115 (PMC12452332; doi:10.3390/geriatrics10050115)
Supplement: Supplementary file 1 [file geriatrics-10-00115-s001.zip › geriatrics-3726237-supplementary.pdf]

Supplementary Information

- Indices of higher-level functional capacity (TMIG-IC, JST-IC)

Article title

Comparison of higher-level functional capacity between older adults with and without type 2 diabetes mellitus: A cross-sectional study using propensity score matching

Journal name

Geriatrics

Author names and affiliations

Takuro Shoji, PT, MS [1, 2], Kenta Kogure, PT [1], Nagisa Toda, PT [1], Mariko Hakoshima, MD [3], Hisayuki Katsuyama, MD, PhD [3], Hidekatsu Yanai, MD, PhD [3], Satoshi Tokunaga, MS [2], Korin Tateoka, MS [4], Taishi Tsuji, PhD [5], Tomohiro Okura, PhD [5]

Takuro Shoji [1, 2], Kenta Kogure [1], Nagisa Toda [1], Mariko Hakoshima [3], Hisayuki Katsuyama [3], Hidekatsu Yanai [3], Satoshi Tokunaga [2], Korin Tateoka [4], Taishi Tsuji [5, 6], and Tomohiro Okura [6]

[1] Department of Rehabilitation Medicine, National Center for Global Health and Medicine Kohnodai Hospital, Ichikawa, Chiba 272-8516, Japan
[2] Graduate School of Comprehensive Human Sciences, Doctoral Program in Public Health, University of Tsukuba, Tuskuba, Ibaraki 305-8574, Japan
[3] Department of Diabetes, Endocrinology, and Metabolism, National Center for Global Health and Medicine, Kohnodai Hospital, Ichikawa, Chiba 272-8516, Japan
[4] Graduate School of Comprehensive Human Sciences, Doctoral Program in Physical Education, Health and Sport Sciences, University of Tsukuba, Tuskuba, Ibaraki 305-8574, Japan
[5] Center for Preventive Medical Science, Chiba University, Chiba 263-8522, Japan
[6] Institute of Health and Sport Sciences, University of Tsukuba, Tuskuba, Ibaraki 305-8574, Japan

Corresponding author

Takuro Shoji, PT, MS
Department of Rehabilitation Medicine,
National Center for Global Health and Medicine, Kohnodai Hospital,

1-7-1, Ichikawa, Chiba 272-8516, Japan
Tel: +81 47-372-3501
Fax: +81 47-375-4746
Email: pt.shoji@hospk.ncgm.go.jp
ORCID: 0000-0002-8471-526X

The Tokyo Metropolitan Institute of Gerontology Index of Competence (TMIG-IC)

1. Can you use public transportation (bus or train) by yourself?
2. Are you able to shop for daily necessities?
3. Are you able to prepare meals by yourself?
4. Are you able to pay bills?
5. Can you handle your own banking?
6. Are you able to fill out forms for your pension?
7. Do you read newspapers?
8. Do you read books or magazines?
9. Are you interested in news stories or programs dealing with health?
10. Do you visit the homes of friends?
11. Are you sometimes called on for advice?
12. Are you able to visit sick friends?
13. Do you sometimes initiate conversations with young people?

The Japan Science and Technology Agency Index of Competence (JST-IC)

1. Can you use a mobile phone?
2. Can you use the ATM?
3. Can you operate a video recorder such as a Blu-ray recorder or DVD player?
4. Can you send an e-mail using a mobile phone or computer?
5. Are you interested in news and events from overseas?
6. Can you determine the credibility of health-related information?
7. Do you enjoy art, films, or music?
8. Do you watch educational/cultural programs?
9. Do you follow any measures to prevent yourself from becoming a victim of crimes.
10. Do you try to be creative while doing daily tasks (i.e., cleaning, cooking)?
11. Can you take care of an ill person?
12. Do you take care of your grandchildren, family members, or acquaintances?
13. Do you participate in regional festivals or events?
14. Do you participate in a neighborhood association or a residents' association?
15. Would you be able to assume a managerial position such as an organizer in a residents' association or group activities?
16. Do you engage in charity or volunteer activities?

Supplementary Figure S1. Indices of higher-level functional capacity (TMIG-IC, JST-IC)
